# Intra- and Inter-observer Variability of Computed Tomographic Measurements of the Prostate Gland in Neutered Dogs

**DOI:** 10.3389/fvets.2021.606116

**Published:** 2021-06-07

**Authors:** Alessandro Delaude, Bart J. G. Broeckx, Jimmy H. Saunders, Lauren De Winter, Amber Hillaert, Emmelie Stock

**Affiliations:** ^1^Department of Veterinary Medical Imaging and Small Animal Orthopaedics, Faculty of Veterinary Medicine, Ghent University, Merelbeke, Belgium; ^2^Department of Nutrition, Genetics and Ethology, Faculty of Veterinary Medicine, Ghent University, Merelbeke, Belgium

**Keywords:** CT, canine, prostate, size, neutered

## Abstract

The purpose of this study was to evaluate the intra- and inter-observer variability of computed tomographic measurements of linear prostate dimensions in neutered dogs without signs of prostatic disease, to determine potential associations between prostatic parameters and body weight or age and to provide reference ranges. Length, width and height of the prostate gland were measured in 62 neutered dogs with no signs of prostatic disease by three observers with different levels of training. Statistically significant positive associations were found between all prostatic parameters and body weight and between all prostatic parameters and age at castration, but not with age. Formulae allowing the calculation of the expected values for prostatic parameters based on body weight are provided [length = 15.3 + body weight (BW) × 0.3; height = 9.7 + BW × 0.16; width = 9.5 + BW × 0.2]. These may represent a useful tool for computed tomographic evaluation of the size of the prostate in neutered dogs. Subjective evaluations of the morphological appearance of the prostate gland are also provided.

## Introduction

The prostate gland is the only accessory gland in male dogs. Its size can be influenced by a wide variety of processes, leading to both increased and decreased sizes. Following castration, the canine prostate for example undergoes an involution as a result of androgen deprivation (1), which leads to a consistently smaller prostate in neutered dogs compared to entire dogs (2–5). On the other hand, prostatic enlargement can be an unspecific sign of prostatic diseases as benign prostatic hyperplasia (BPH), prostatitis, or prostatic neoplasia (6). BPH occurs mostly in intact male dogs, since the prostate exhibits continual, androgen-dependent growth (7). BPH predisposes to prostatitis, which, on the contrary, has rarely been reported in neutered male dogs (8). Instead, neutered dogs are predisposed to developing prostate tumors, in particular transitional cell carcinoma (TCC) (9). Prostatic involvement in case of multicentric lymphoma leading to prostatomegaly has been recently described as well (10). Age of neutering (early or late) and the presence of previous disease can also influence the size of the prostate. According to previous literature (2), the prostate might even not be delineable on CT images in some neutered dogs.

To the authors' knowledge, reference ranges for normal prostate size in neutered dogs have not been published yet. Thus, the evaluation of the prostate in neutered dogs is quite subjective and sometimes difficult. The use of Computed Tomography (CT) in veterinary medicine has become more and more frequent, in line with reduced costs and an increased availability of teleradiology services. Due to the intrapelvic location of the prostate and some of its draining lymph nodes and to the possible concurrent involvement of the surrounding skeletal structures in case of neoplastic disease (11), CT examination can be a particularly helpful diagnostic tool to guide clinical decisions (12). Here, we aim to describe the normal anatomy of the prostate gland in neutered dogs, determine the intra- and inter-observer variability of CT-measurements of the size of the prostate gland, provide reference ranges for prostate size and evaluate the influence of age and body weight on the size of the prostate gland in neutered dogs.

## Materials and Methods

### Patient Selection

In this retrospective study, CT images of mature, neutered male dogs with no reported clinical signs of prostatic disease that underwent CT examination of the pelvic region between 2016 and 2020 at the University Small Animal Hospital of the Faculty of Veterinary Medicine of Ghent (Belgium) were retrieved. The CT scans were only included if the entire prostate, the urinary bladder and part of the pelvic urethra caudal to the prostate were included in the field-of-view. Exclusion criteria were prostate incompletely included in the field-of-view, poor quality studies (presence of artifacts), and indications of prostatic pathology based on anamnesis and clinical examination (Table 1). When not mentioned in the clinical history, owners of the dogs included in the study were contacted telephonically to ask at which age their dogs were neutered.

**Table 1:**
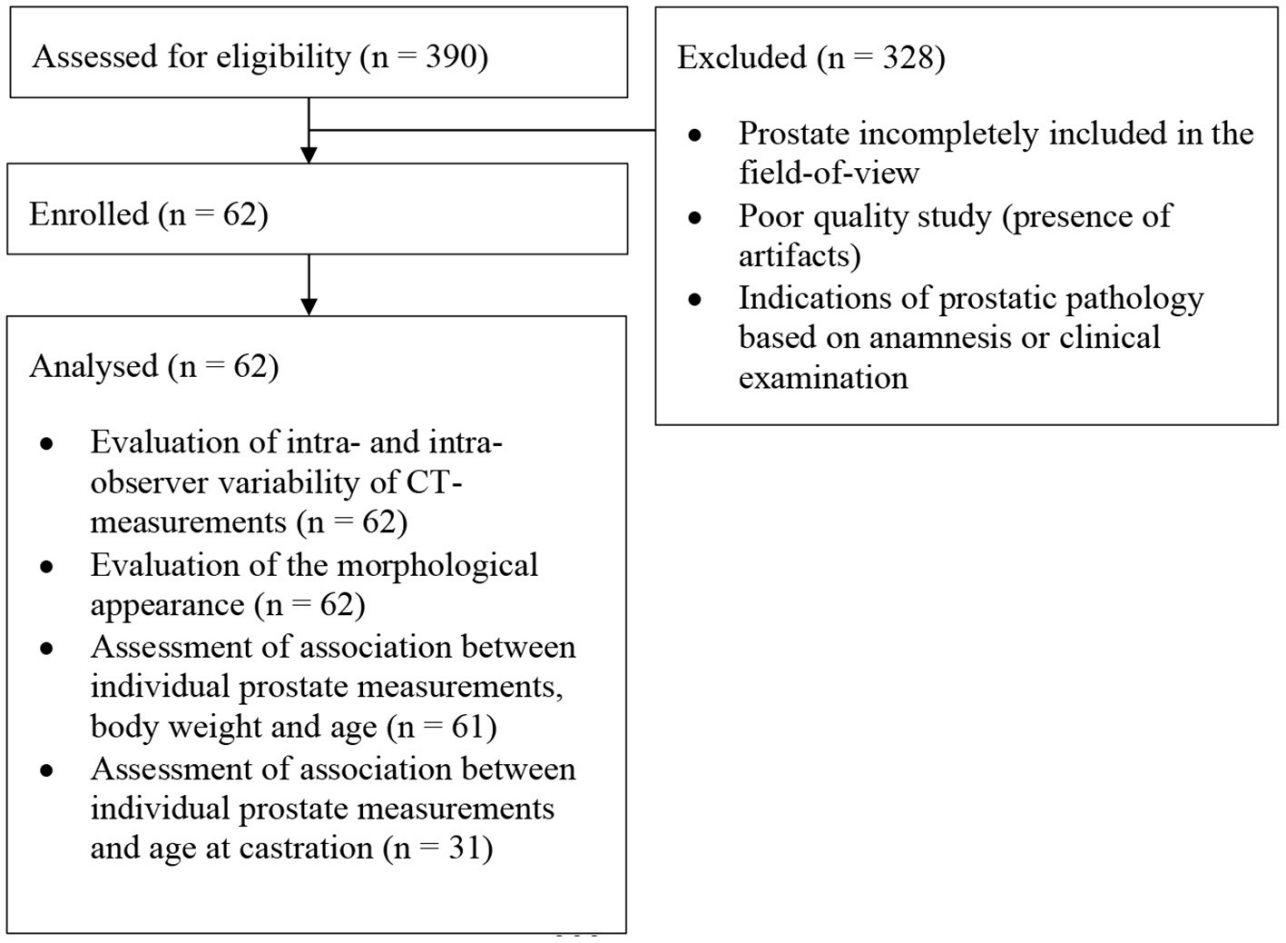
CONSORT diagram.

### Image Acquisition Technique

A four-slice helical CT device (GE Lightspeed QX/I; General Electric Co., Milwaukee, MI) was used for all scans with the following acquisition variables: voltage = 120 kVp; current = 120–140 mA; beam pitch: 0.75; slice width: 1.24–2.5 mm. Images were reconstructed with a soft-tissue algorithm. Both native images and images acquired after intravenous injection of iodinated contrast medium were available for the majority of the patients (53/62), whereas either native (5) or post-contrast images (4) were available for the remaining dogs. When a contrast study was performed, 2 ml/kg of iodinated, low osmolar non-ionic contrast medium at a concentration of 300 mg I/ml (iohexol, Omnipaque^TM^) or 400 mg I/ml (iomeprol, Iomeron®) was manually administered IV and images were acquired in a venous or late parenchymal phase, ~50–120 s after injection. Dogs were positioned on the scanning table in dorsal or ventral recumbency under general anesthesia or deep sedation depending on the clinical indications.

### Image Evaluation

Observers with different backgrounds in radiology, namely a board-certified radiologist (E.S.), a radiology resident (A.D.) and a PhD student (A.H.), examined the CT-images three non-consecutive times and measured prostatic length, height and width. The PhD student attended a 15-min training to identify and measure the prostate under the guidance of the radiology resident (A.D.).

**Figure 1:**
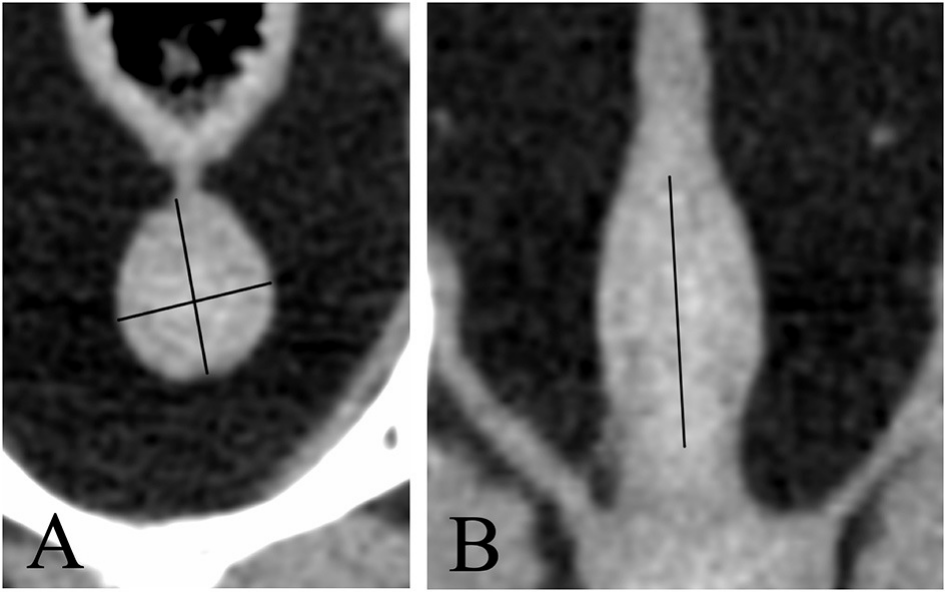
Transverse **(A)** and dorsal **(B)** CT-reconstruction showing the prostate of a neutered dog. Length, height, and width are measured (black lines).

**Figure 2:**
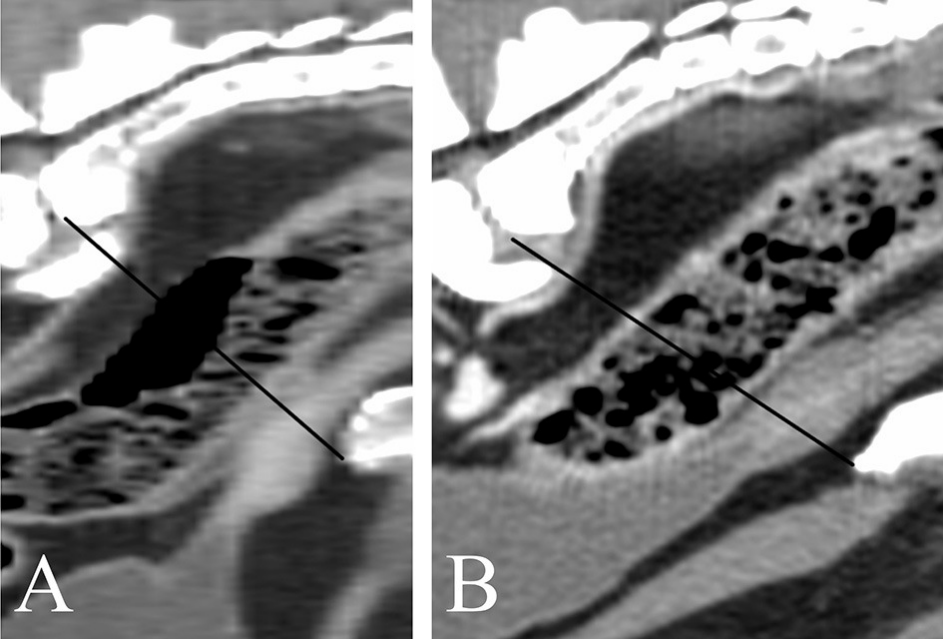
Sagittal CT-reconstruction showing prostates in an abdominal **(A)** and pelvic location **(B)**, respectively. The black line indicates the pelvic brim.

Images were reviewed using commercially available viewing software (OsiriX, v.10.0.2.; Pixmeo SARL, 226 Rue de Bernex, CH1233 Bernex, Switzerland). Length was defined as the maximum diameter of the gland along the urethral axis. Height was defined as the maximum diameter perpendicular to the axis of the length. Width was defined as the maximum diameter perpendicular to the axis of the height on transverse images (Figure 1). Morphological features including shape, symmetry, pre- and post-contrast homogeneity, lobe margins, visualization of median septum, urethral enhancement, and urethral shape, determined in consensus by the radiology resident (A.D.) and the board-certified radiologist (E.S.). The anatomical location of the prostate and its relation to the filling status of the urinary bladder were evaluated as well; the prostate was considered abdominal when more than 50% was located cranial to a line extending from the cranioventral margin of the os sacrum to the pecten of pubic bone, corresponding to the pelvic brim, and pelvic when more of 50% of it lay caudal to this line (Figure 2). The filling status of the urinary bladder was classified as poor, moderate or severe based on size, wall tension and mass effect against the adjacent abdominal organs.

### Statistical Analysis

The statistical analysis was done in R version 3.6.3 (“Holding the Windsock”). Significance was set at α ≤ 0.05. Descriptive statistics are provided as mean and standard deviation or median and range after normality checks with QQ-plots were performed. To evaluate the variability, a random effects model was specified with animal and person within animal as random effects. The variance components were estimated with restricted maximum likelihood. The residual variance is a measure of the intraobserver variability, while the added variance due to person provides a measure of the interobserver agreement. Finally, the added variance due to animal gives an idea of the variability when different animals are considered. These three variance components were used to calculate the 95% intraobserver limits of agreement, the 95% interobserver limits of agreement and the 95% reference interval for different animals. A mixed model with weight, age or age of castration as fixed effect and animal and person within animal as random effect was used to assess the potential association between one of the fixed effects and the individual prostate measurements (as dependent variable). A likelihood ratio test was used to evaluate the significance of the fixed effect. Finally, the expected values, based on an earlier report (13), and the observed values were compared with a paired Student's *t*-test and Bland–Altman curves.

## Results

Based on the inclusion criteria, 62 mature, male neutered dogs were included in this study. The dogs were between 13 months and 14 years old with a mean of 7.8 years of age (standard deviation: 38 months). The age at castration was known in 31/62 dogs; the median age at castration was 18 months (4–132 months); 19/31 dogs were castrated at or before 18 months of age; 2/31 dogs were castrated at 24 and 36 months, respectively, 10/31 dogs were castrated at or after 48 months of age. 2/31 dogs, both neutered before 12 months of age, were castrated 3 and 4 months before the CT scan, respectively, whereas all the remaining dogs were castrated at least 12 months before the images were acquired. The body weight was known in 61/62 patients and varied between 3.8 and 59 kg with a mean of 26 kg and a standard deviation of 16 kg. A similar number of small, medium and large dogs was included: 22 dogs weighing up to 15 kg, 15 weighing between 15 and 30 kg and 24 weighing more than 30 kg.

The most frequent breed included was mixed breed (12.7%) followed by Malinois (7.9%), Jack Russel Terrier (7.9%), English Cocker (6.3%), Dachshund (4.8%), Labrador Retriever (4.8%), Bernese Mountain Dog (4.8%), and Golden Retriever (3.2%).

The prostate was visible in 62/62 neutered dogs. Overall, the smallest intra- and inter-observer 95% limits of agreement were found for the prostatic width (respectively, ±1.7 mm and ±2.2 mm), followed by height (±2.9 mm and ±1.8 mm) and length (±6.7 mm and ±3.7 mm; Table 2). The mean length of the prostate [±standard deviation (SD)] was 23.2 (±7.0) mm; the mean (±SD) height and width measured, respectively, 13.9 (±4.4) mm and 14.9 (±5.6) mm. The 95% reference ranges are also provided in Table 2.

**Table 2:** SD, standard deviation; LoA, limits of agreement; into brackets: 95% reference interval.

	**Length**	**Height**	**Width**
Intraobserver SD	1.9 mm	0.9 mm	0.8 mm
Intraobserver LoA	±3.7 mm (19.5–26.9 mm)	±1.8 mm (12.1–15.7 mm)	±1.7 mm (13.2–16.5 mm)
Interobserver SD	2.9 mm	1.2 mm	0.7 mm
Interobserver LoA	±6.7 mm (16.5–30 mm)	±2.9 mm (11–16.8 mm)	±2.2 mm (12.7–17 mm)

A significant association was found between each prostatic parameter and the body weight (*P* < 0.001) and between each prostatic parameter and the age of the dog at castration (length: *P* < 0.05; height and width: *P* < 0.001), but not between the prostatic parameters and the age of the patient (*P* > 0.05). The relation between body weight (BW) and the maximum expected values for length, height, and width of the prostate expressed in millimeters can be calculated with the following formulae:

$$\begin{array}{l} \text{Length}=\text{15}.\text{3}+\text{BW}\times 0.\text{3}\\ \text{Height}=\text{9}.\text{7}+\text{BW}\times 0.\text{16}\\ \text{ Width}=\text{9}.\text{5}+\text{BW}\times \;0.\text{2} \end{array}$$

**Figure 3:**
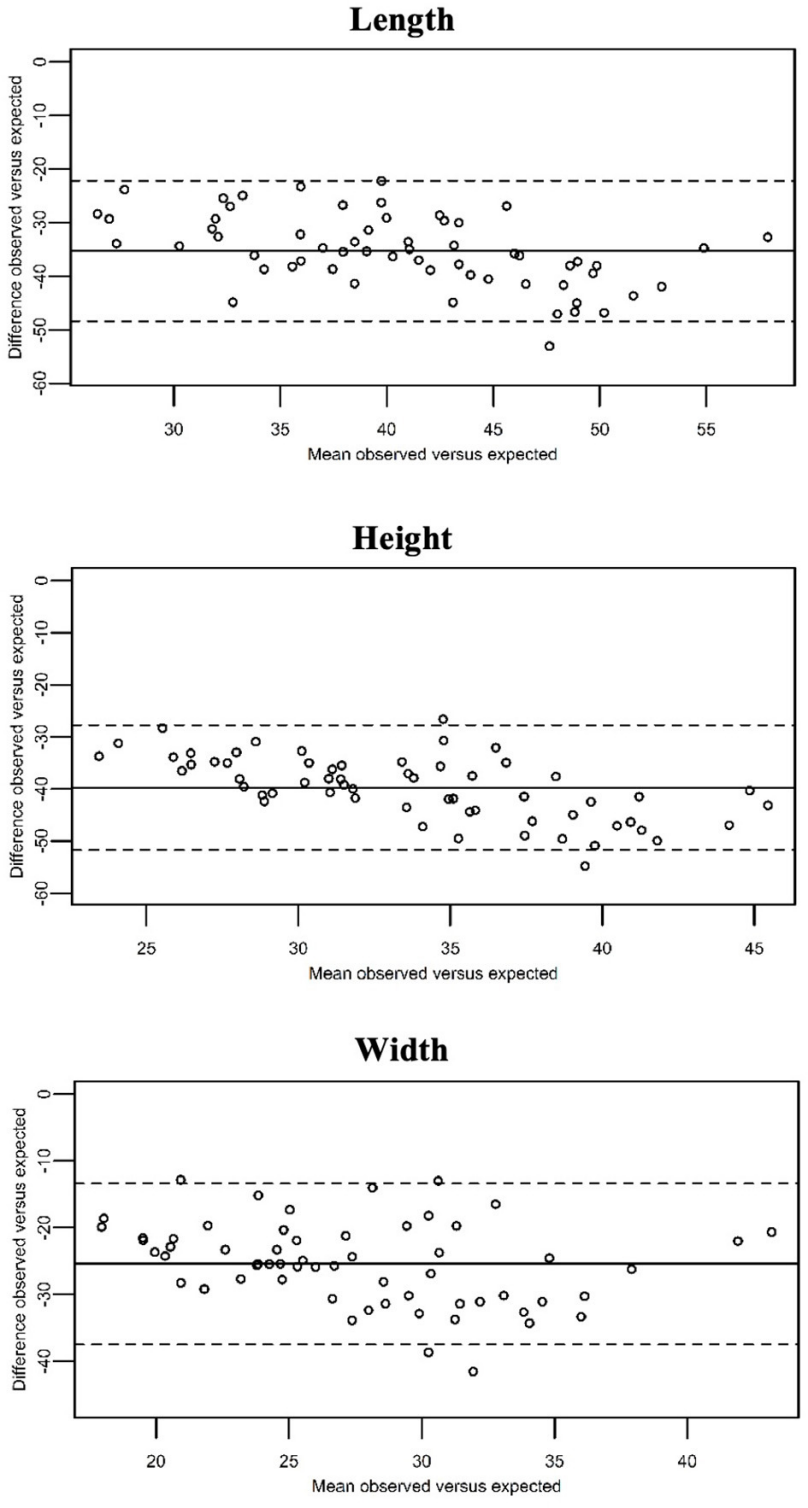
Bland–Altman curves showing the mean of the observed prostatic dimensions in our population of neutered dogs vs. the expected values (x-axis), plotted against the difference between these and the expected prostatic dimension in entire dogs (y-axis). The expected values were calculated by means of the formulas provide by Ruel et al. The continuous line represents the overall mean difference between the observed and the expected prostatic dimensions. As the observed prostatic size increases, the difference between the observed and the expected prostatic dimensions increases as well.

The observed values differed significantly from the expected maximum predicted prostatic dimensions of entire dogs calculated according to the formula of Ruel et al. (*P* < 0.01; Figure 3).

**Figure 4:**
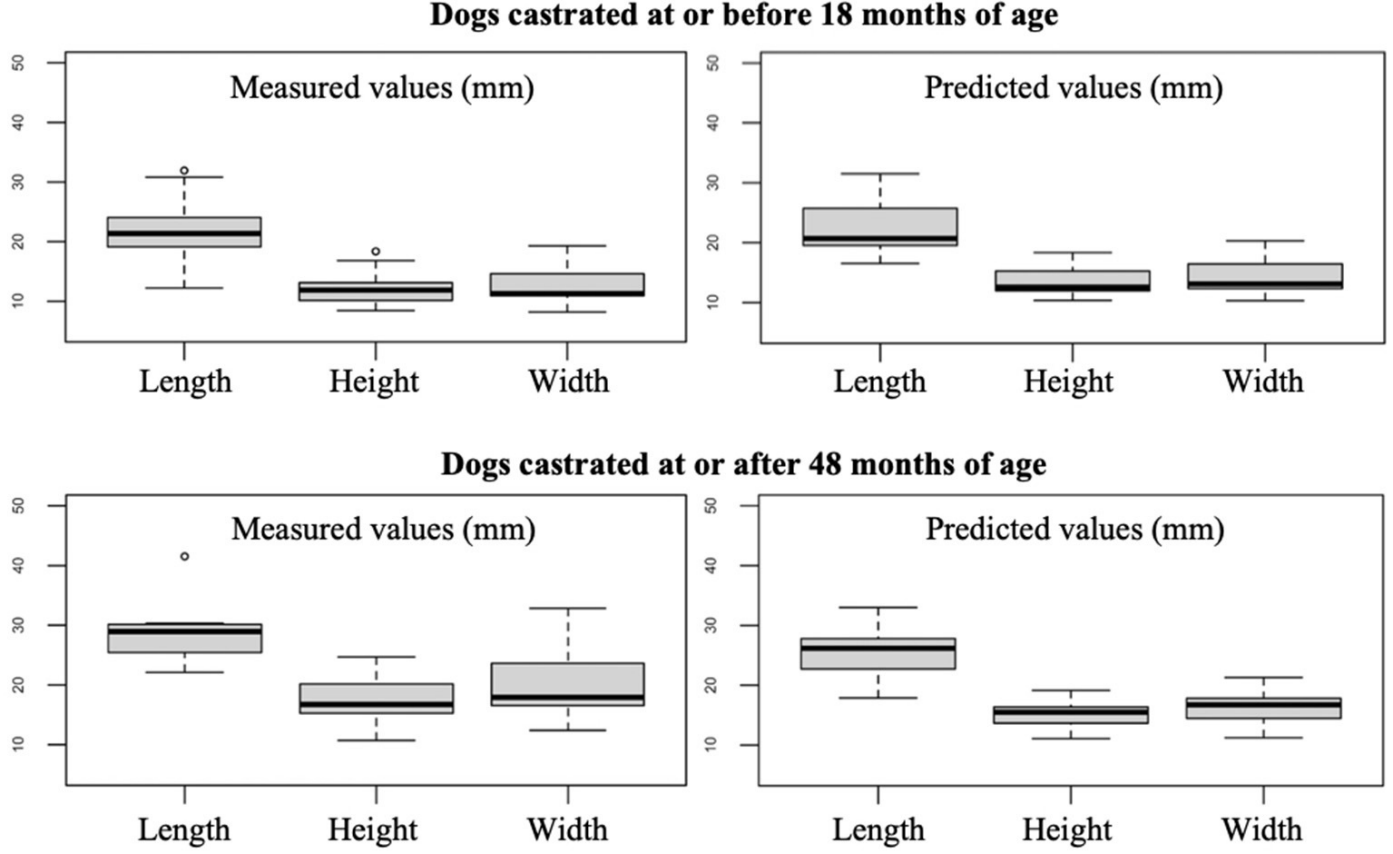
Box plots showing the measured and predicted prostate measurements for dogs castrated at or before 18 months of age and at or after 48 months of age. The two dogs castrated 3 and 4 months before undergoing the CT scan were not included.

The prostate size of dogs castrated at or before 18 months of age was smaller than that of dogs castrated at or after 48 months of age, having a median length, height and width of 21, 11.6, and 12 mm vs. 27.9, 17, and 18.5 mm, respectively. The predicted prostate measurements for dogs castrated at or after 48 months of age were slightly smaller than that observed values (median length, height, and width of 26.2, 15.5, and 16.7 mm, respectively; Figure 4).

**Figure 5:**
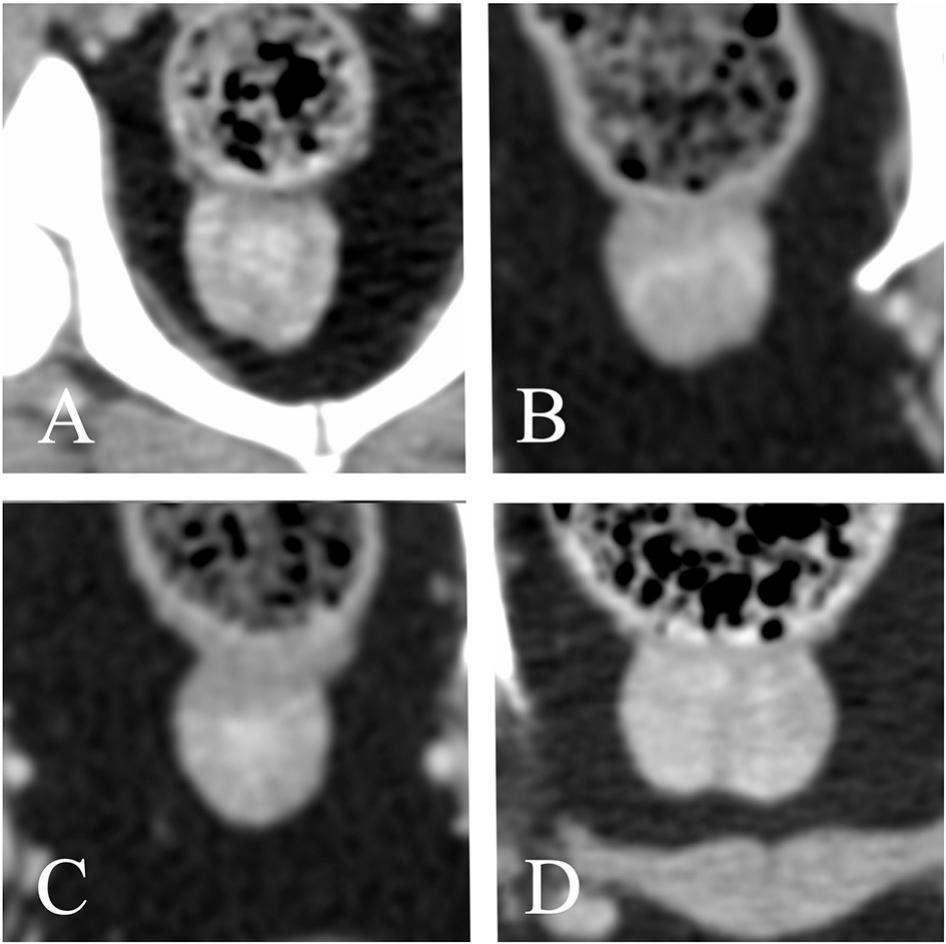
Transverse reconstructions of the prostate showing different pattern of contrast enhancement. **(A)** Peripheral enhancement. **(B–D)** Heterogeneous, randomly distributed contrast enhancement. **(A,C)** urethral enhancement. **(A,B,D)** A shallow notch in the ventral surface of the prostate marks the boundary between right and left lobe. **(B)** Slightly asymmetrical prostate. **(C)** No ventral notch allowing a clear distinction between right and left lobe is noted. **(D)** A prominent median septum is visible.

The anatomical location of the prostate was abdominal in 18/62 dogs (29%) and pelvic in 44/62 dogs (71%). Considering only the dogs with known age at castration, the anatomical location of the prostate was mostly pelvic in both dogs castrated at or before 18 months of age (14/19, 73.7%) and dogs castrated at or after 48 months of age (6/10, 60%). The urinary bladder was considered moderately filled in most of the patients (40/62, 57.2%), large in 15/62 dogs (21.7%), and small in the remainder (7/62, 10.1%). Among dogs with an abdominal prostate, 2/18 (11.1%), 12/18 (66.7%), and 4/18 (22.2%) patients showed a poorly, moderately, and severely filled urinary bladder, respectively. 5/44 (11.1%), 28/44 (63.6%), and 11/44 (25%) dogs with a pelvic prostate showed a poorly, moderately, and severely filled urinary bladder, respectively. The shape of the prostate was ovoid in most patients, with only one dog presenting a cone-shaped prostate with the caudal pole wider than the cranial pole. A distinct separation between the prostatic lobes recognizable as a shallow notch in the ventral aspect of the gland was visible in 30/62 (49.1%) dogs (Figures 5A,B,D). The lobes were symmetrical in most dogs (59/62, 95.2%); 3/62 dogs (4.8%) showed lobes with slightly different size and sideward displacement of the notch marking their boundary (Figure 5B). A prominent median septum was observed on post-contrast images as a poorly enhancing band in the midline in 18/57 dogs (31.6%) (Figure 5D).

**Figure 6:**
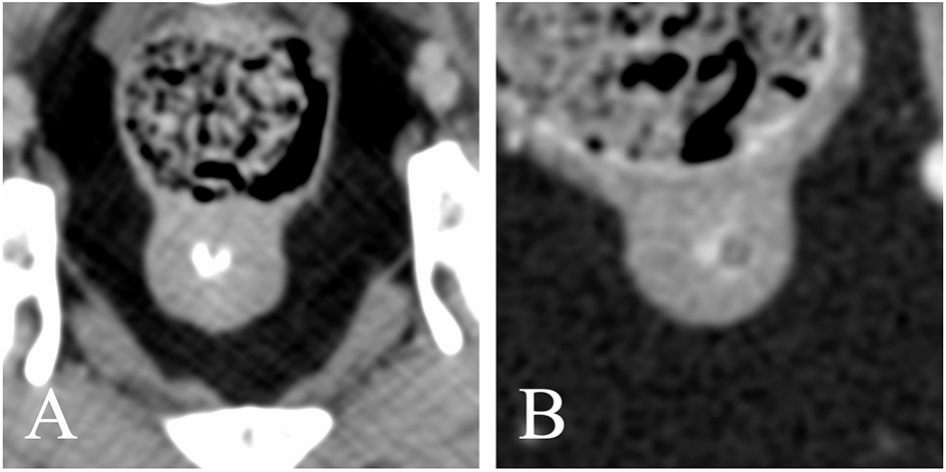
Transverse reconstructions showing the different shape of the intra-prostatic urethral lumen **(A)** V-shaped; **(B)** O-shaped. Note the marked urethral enhancement in panel **(B)**.

A native study was available for 58/62 dogs. A contrast study was available for 57/62 dogs. Both pre- and post-contrast images were available for 53/62 dogs. All prostates appeared homogeneous in non-contrast CT images. 50/57 prostates (12.3%) showed homogenous contrast enhancement. One hundred percent of dogs castrated before 18 months of age with available contrast study (18/19) demonstrated homogeneous contrast enhancement of the prostate, whereas only 55.6% (5/9) of dogs castrated after 48 months had a homogeneously contrast enhancing prostate. When heterogeneous, either stronger peripheral enhancement (2/7) or ill-defined, stronger contrast enhancing areas with random distribution (5/7) were noted (Figure 5). Twenty-eight dogs (49.1%) showed urethral enhancement. The shape of the prostatic urethra on transverse images was discernable in 22/62 dogs (35.5%): the urethra was “V” shaped in 19/22 patients (86.4%) and “O” shaped in 3/22 patients (13.6%) (Figure 6).

## Discussion

The main goals of this study were to provide an indication of the intra- and inter-observer variability of CT-measurements of the prostate in neutered dogs performed by veterinarians with different levels of training and to provide reference values for the normal prostate size in neutered dogs.

Overall, the SD and LoA of the CT-measurements were sufficiently small (Table 2). Slightly higher SD and LoA were noted for the length, compared to height and width. Atalan et al. and Lee et al. recommend the use of width and length to estimate the actual prostate size in entire dogs. According to Atalan et al., these parameters, measured both physically at necropsy or ultrasonographically, were the best predictors for prostatic volume and weight. In the study of Lee et al., width and length showed the best correlation with the prostatic area. We found the best inter- and intra-observer agreement for the width. The findings of our study combined with the observations of Atalan et al. and Lee et al. suggest that the width could be the most useful parameter to estimate the prostate size in neutered dogs.

CT-measurements of the prostate in healthy entire dogs are similar to the ultrasonographic measurements (14), therefore we compared the observed measurements to the expected prostate size in entire dogs, calculated with the formulae provided by Ruel et al. As reported in previous studies (2–4), we confirmed that neutered dogs have a significantly smaller prostate gland compared to entire dogs.

Up to date, there is limited data about the CT evaluation of the prostate gland in neutered dogs. Atalan et al. included 17 neutered dogs in their study, Dirrig et al. 23 and Haverkamp et al. 37, but none of them provides formulae to estimate the prostate size in neutered dogs. Therefore, following the example of Ruel et al., we developed formulae to predict the expected value of length, height, and width of the prostate. Since we did not find any significant correlation between prostate size and age, as already observed by Atalan et al. and Haverkamp et al., we considered only body weight as a variable. The clear correlation between the prostatic size and the body weight is in agreement with previous studies (5, 13, 14) and reflects the necessarily close correlation between the size of a given organ and the size of the organism of which it forms a part; furthermore, obesity and fatty infiltration of the prostate might play a role as well. The age at castration showed a significant association with the prostate size, with the dogs castrated at an older age demonstrating slightly larger prostates than predicted by the provided formulae (Figure 4). However, since the age at castration was known in a limited number of patients only and considering that most of them were neutered before 18 months of age, we decided not to include the age at castration as a variable in the formulae. Interestingly, the two dogs which were castrated <4 months before the acquisition of the images showed a prostate size similar to the predicted values, with only the prostate width being above the predicted value (14.5 and 16 mm vs. 11.3 and 14 mm, respectively). This suggests that the involution of the prostate happens quite fast within a few months after castration.

None of the dogs included in our study had clinical signs compatible with prostatic disease or evidence of prostatic disease on CT-images, such as masses, intra- or paraprostatic cysts or surrounding steatitis; therefore, we presumed that their prostates were normal. Since the prostatic lobes were similar to each other in most of our patients, with only three prostates being slightly asymmetrical, we measured the overall prostatic height without differentiating between left and right lobe. The asymmetry was represented by a mild sideward displacement of the ventral notch demarcating the boundary between lobes (Figure 5B). No obvious cause was visible explaining the asymmetry; we therefore suppose that this could represent a normal anatomical variant or be secondary to a previous prostatic disease. Unfortunately, no histopathological examination was available to corroborate our assumption. Another limitation is the fact that the age at castration and the time elapsed between the castration and the CT-examination is unknown in half of the patients included and this could affect the appearance and size of the prostate.

According to Dimitrov et al. (15), the prostate is originally situated in the pelvic cavity and it progressively moves into the abdominal cavity secondary to sexual maturation and hormonally induced hyperplasia. In the study of Pasikowska et al., the prostate was situated in the pelvic cavity in all dogs with no evidence of prostatic hyperplasia, whereas 30% of the hyperplastic prostates were located in the abdominal cavity. In our study, 18 prostates (29%) were located cranial to the pelvic brim. A possible explanation is that these dogs were castrated in their adulthood, after the migration of the prostate into the abdominal cavity, and that it did not return in a pelvic position; a slightly larger proportion of dogs castrated after 48 months of aged had an abdominal prostate, compared to dogs castrated before 18 months of age (40%, 4/10, vs. 26.3%, 5/19). In our population, the abdominal and pelvic prostate groups showed a similar proportion of dogs with poorly, moderately and severely filled urinary bladder; thus, the impact of the filling status of the urinary bladder on the anatomical position of the prostate might be limited.

Dirrig et al. did not identify the prostate in 2/23 neutered dogs, whereas it was visible in all our patients, appearing, when particularly atrophic, as a slight, focal, circumferential increase in thickness of the proximal urethra, best seen on dorsal and sagittal reconstructions. The abundant surrounding fat provides good contrast between the prostate and the adjacent organs. Dirrig et al. could recognize distinct lobes in only 5/21 patients (23.8%), whereas we could identify them in 30/62 dogs (49.1%). On transverse image, the separation between lobes was demarcated by a shallow notch on the midline, best seen in the ventral surface, which tended to flatten or disappear in the smallest prostates (Figures 5B,C). This notch was less evident in the dorsal surface, since the prostate was in direct contact with the ventral wall of the rectum in all patients. In 18/57 dogs (31.6%), a prominent poorly enhancing septum was noted on post-contrast images, allowing clearer distinction between right and left lobe (Figure 5D). The mean prostatic dimensions of our population are similar but slightly larger compared to those reported in the study of Dirrig et al. (length: 20 mm; height 12 mm; width: 15 mm). A possible explanation for the discrepancy between their findings and ours, is that a larger number of dogs with extremely atrophic prostate was included in the study of Dirrig et al., thus making the delineation of the prostate more difficult.

On native images, the prostate was homogeneous in all patients; mild heterogeneity was observed in seven dogs (12.3%) after application of contrast medium. 4/7 of these heterogeneously contrast enhancing prostates belonged to dogs castrated after 48 months of age, whereas all dogs castrated before 18 months of age showed homogeneous contrast enhancement, suggesting a potential role of age at castration in the enhancement pattern of the prostate. Parenchymal heterogeneity is often due to prostatic cysts, intraparenchymal mineralization, and possibly heterogeneous blood flow, which are commonly observed in entire dogs affected by clinical or subclinical benign prostatic hyperplasia (6, 12, 14), but rarely in castrated dogs (2, 8). Dirrig et al. found only 1/23 neutered dog with heterogeneous prostate on native images; he had been castrated recently, therefore he was supposedly still undergoing prostatic involution. Of the two dogs in our sample that had been castrated less than 4 months before the acquisition of the images, only one received contrast medium and showed homogeneous contrast uptake in the prostate. Since all the prostates showing mild heterogeneous contrast enhancement were homogeneous in the native study and no distinct cystic structures were recognizable, we presume that the main reason for heterogeneous contrast enhancement in our patients is related to the blood perfusion of the prostate and to the timing between the application of the contrast medium and the acquisition. Most of the contrast-studies reviewed were acquired during the systemic venous phase, but the scanning-protocol was not standardized, since the contrast medium was injected manually in most of the patients. Therefore, the actual phase may play an important role in the determination of the contrast enhancement pattern of the prostate. Dirrig et al. too found 3/21 neutered dogs having homogeneous prostatic attenuation pre-contrast and heterogeneous contrast enhancement.

As previously described by Dirrig et al., the prostatic urethra may demonstrate ring-like enhancement and appear V- or O-shaped. The urethral lumen was visible in 22/62 dogs and best seen when filled with contrast medium; it appeared V-shaped in 19 dogs (86.4%), presumably due to the presence of the urethral crest, a short longitudinal fold on the dorsal wall of the prostatic part of the pelvic urethra (16). In three dogs, the urethral lumen appeared rather O-shaped; one dog showed a diffusely dilated pelvic urethra; the other two had a poorly filled prostatic urethra with narrow lumen.

Prostatic enlargement is a non-specific abnormality which may be caused by a variety of inflammatory, non-neoplastic and neoplastic diseases (9, 17, 18). Considering that only a small percentage of dogs with a non-malignant disorder are castrated (19), while neutering status might represent a risk factor for prostate cancer (9, 19, 20), prostatic enlargement in castrated dogs should be regarded as suspicious for malignancy and therefore evaluated carefully (18).

In conclusion, CT examination is a viable method to estimate the prostate size in neutered dogs. Formulas to predict the expected prostate size in neutered dogs are provided. These may represent an additional tool to help guiding clinical decisions. Further research is necessary to determine the extent of the effect of age at castration and time elapsed between castration and CT-examination on prostate size.

## Data Availability Statement

The original contributions presented in the study are included in the article/supplementary material, further inquiries can be directed to the corresponding author/s.

## Ethics Statement

Ethical review and approval was not required for the animal study because it is a retrospective study based on images acquired for clinical reasons, which were totally independent from the purpose of this research. Written informed consent for participation was not obtained from the owners because Clients visiting the University Small Animal Hospital of the Faculty of Veterinary Medicine of Ghent are orally informed that the data acquired could be used anonymously for research.

## Author Contributions

AD, JS, and BB: conception and design. AD and BB: drafting the article. AD, ES, BB, AH, and LD: analysis and interpretation of data. AD, ES, BB, JS, AH, and LD: revising article for intellectual content, and final approval of the completed article. All authors contributed to the article and approved the submitted version.

## Conflict of Interest

The authors declare that the research was conducted in the absence of any commercial or financial relationships that could be construed as a potential conflict of interest.
